# Role of Renal Parenchyma Attenuation and Perirenal Fat Stranding in Chest CT of Hospitalized Patients with COVID-19

**DOI:** 10.3390/jcm12030929

**Published:** 2023-01-25

**Authors:** Elisa Russo, Alberto Stefano Tagliafico, Lorenzo Derchi, Bianca Bignotti, Simona Tosto, Carlo Martinoli, Alessio Signori, Francesca Brigati, Francesca Viazzi

**Affiliations:** 1 Nephrology Unit, Ospedale San Luca, 55100 Lucca, Italy; 2 Department of Health Sciences (DISSAL), University of Genova, 16126 Genova, Italy; 3 Radiologic Unit, IRCCS Ospedale Policlinico San Martino, 16132 Genova, Italy; 4 Department of Experimental Medicine (DIMES), University of Genova, 16126 Genova, Italy; 5 Department of Internal Medicine, University of Genoa, IRCCS Ospedale Policlinico San Martino, 16132 Genova, Italy

**Keywords:** SARS-CoV-2, COVID, computed tomography, acute kidney injury, renal parenchyma attenuation, perirenal fat stranding, mortality risk

## Abstract

Background: Chest CT on coronavirus disease (COVID-19) has been extensively investigated. Acute kidney injury (AKI) has been widely described among COVID patients, but the role of kidney imaging has been poorly explored. The aim of this study is to clarify the role of opportunistic kidney assessment on non-enhanced chest CT. Methods: We collected data on patients with COVID-19 consecutively admitted to our institution who underwent chest CT (including the upper parts of kidneys as per protocol). Three ROIs of 0.5–0.7 cm^2^ were positioned in every kidney. The values of renal parenchyma attenuation (RPA) and the presence of perirenal fat stranding (PFS) were analyzed. The primary and secondary outcomes were the occurrence of AKI and death. Results: 86 patients with COVID-19 and unenhanced chest CT were analyzed. The cohort was split into CT RPA quartiles. Patients with a CT RPA <24 HU were more likely to develop AKI when compared with other patients (χ^2^ = 2.77, *p* = 0.014): at multivariate logistic regression analysis, being in the first quartile of CT RPA was independently associated with a four times higher risk of AKI (HR 4.56 [95% CI 1.27–16.44, *p* = 0.020). Within a mean 22 ± 15 days from admission, 32 patients died (37.2%). Patients with PFS were more likely to die as compared to patients without it (HR 3.90 [95% CI 1.12–13.48], *p* = 0.031). Conclusions: Detection of low RPA values and of PFS in COVID-19 patients independently predicts, respectively, the occurrence of AKI and an increased risk for mortality. Therefore, opportunistic kidney assessment during chest CT could help physicians in defining diagnostic and therapeutic strategies.

## 1. Introduction

Severe acute respiratory syndrome coronavirus 2 (SARS-CoV-2) has spread worldwide since early 2020 and the World Health Organization stated as a pandemic the related syndrome, Coronavirus Disease-2019 (COVID-19), on 11 March 2020 [1]. COVID 19 was first described in Wuhan, Hubei Province, China in the last months of 2019, and the first European region facing a COVID-19 outbreak was Northern Italy [2].

The lungs are the primary organ affected by SARS-CoV-2, but several studies suggested the virus can also affect other organs, firstly the kidney [3,4]. The reported prevalence of kidney involvement during SARS-CoV-2 is high [5,6,7,8,9] and its origin is considered to be multifactorial. ACE2 receptors on kidneys podocytes and proximal convoluted tubules cells could be related to SARS-CoV-2 pathophysiology of acute kidney injury (AKI), possibly due to direct cytopathic effect of the virus [6,9].

Chest CT is a well-known tool in detecting and monitoring lung impairment in COVID-19 patients [10,11,12]. Over the last three years, a very high number of studies (almost 3000) have been published on the diagnostic and prognostic role of chest CT in COVID-19 [13,14].

Despite the kidney being frequently affected during SARS-CoV-2 infection, there are few studies on kidney imaging. Contrast-enhanced CT demonstrated renal vein thrombosis and renal infarctions [15,16] and, with the use of dual-energy techniques, identified areas of heterogeneous enhancement at the renal cortex even in patients with no evidence of abnormal kidney function [14,17]. Furthermore, a formula using blood urea nitrogen (BUN), renal cortex/aorta attenuation ratio and patient gender was found to be predictive of AKI development, thus suggesting a role of reduction in renal flow as a cause of AKI in these patients [17]. Notably, it has been underlined that contrast media should be used with caution in patients affected by COVID-19 due to their possible adverse effects on renal function [18]. Some information can be obtained from unenhanced CT: while looking at the kidneys in the most caudal images of chest CT studies, a decrease in renal parenchymal attenuation (RPA) and presence of perirenal fat stranding (PFS) have been described in COVID-19 patients and a weak linear correlation between low density and increased serum creatinine levels has been observed [19]. This paper suggests a possible relationship between clinical kidney involvement and changes in kidney imaging during SARS-CoV-2 infection.

The purpose of this study is to clarify the role of opportunistic kidney assessment of non-enhanced chest CT in order to evaluate whether “smart measures” might provide relevant clinical information regarding kidney function and patient prognosis in COVID-19.

## 2. Materials and Methods

### 2.1. Patients

This is a retrospective, observational study involving all adult patients (aged ≥18 years) admitted to the Policlinico San Martino Hospital, Genoa, Italy between 12 February 2020, and 15 May 2020 with respiratory sample positive for SARS-CoV-2 by polymerase chain reaction (PCR) who performed a non-enhanced chest CT for clinical reasons. 

Patients able to give informed consent agreed to be included in the study. For patients unable to provide informed consent, due to the observational nature of the study, no risks and no potential adverse effects for the patients, the necessity to have written informed consent was waived. The study was approved by the Ethics Committee of Liguria Region (CER Liguria, 114/2020-ID 10420) and was carried out in accordance with the principles of the Declaration of Helsinki and REporting of studies Conducted using Observational Routinely-collected health Data (RECORD) [20]. The medical records of the patients were collected at admission, including gender, age, prevalence of comorbidity, Charlson score, vital signs and laboratory test results. Moreover, in-hospital antibiotics, antiviral, anti-inflammatory treatments and the need for mechanical ventilation or kidney replacement treatment (KRT) were retrieved from electronic medical records. The estimated glomerular filtration rate (eGFR) was calculated using the Chronic Kidney Disease Epidemiology Collaboration creatinine equation [21]. AKI was defined according to Kidney Disease: Improving Global Outcomes (KDIGO) criteria [22] as an increase in serum creatinine by >0.3 mg/dl or an increase in serum creatinine to >1.5 times baseline. We did not consider the urine output criteria to define AKI because of missing data due to the retrospective nature of the study. AKI was calculated at three different time-points: (a) at hospital admission, comparing creatinine with the median value of serum creatinine calculated from all available values within 180 days before admission, (b) within the first week, and (c) after a week of hospitalization, comparing creatinine with values at admission. The principal and secondary outcomes were the occurrence of AKI, and intra-hospital and 9 months all-causes death.

All patients for whom images of renal parenchyma were visualized at least to the hilum were included and evaluated. Exclusion criteria were: urolithiasis, renal tumors, respiratory artifacts altering kidney attenuation values. For the purpose of the study, all patients who underwent contrast-enhanced CT, mainly to rule-out pulmonary embolism, were not included to avoid differences in attenuation values estimation on kidney parenchyma visualized on CT.

### 2.2. CT Scanning Protocol

CT scans were performed on different scanners depending on hospital equipment availability for COVID-19 patients during the emergency. The equipment used comprised a 128 slice CT-scanner (Somatom Definition Flash, Siemens, Erlangen, Germany), 64-slice CT-scanner (GE Optima, GE Medical Systems, Milwaukee, WI, USA), 16 slice CT-scanner (GE Medical Systems) and 64 slice CT-scanner (Siemens Healthcare). Patients were scanned in supine position, full inspiration, using a standard volumetric protocol for unenhanced chest CT. For the purpose of this study, CT images were reconstructed with 1.25 mm slice thickness and a spacing of 1.25 mm and evaluated using abdominal windowing (30-360 Hounsfield Unit (HU)). The standard chest CT-scan acquired in full inspiration included part of both kidneys as per protocol. 

#### Qualitative Analysis

**Figure 1:**
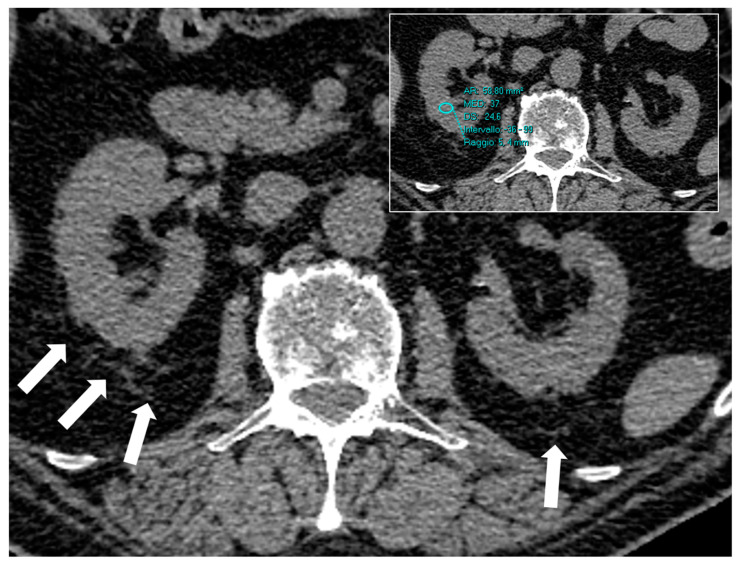
Axial non-enhanced CT of a 65-year-old admitted with COVID-19. CT images show a typical case of perinephric fat stranding (arrows). Renal parenchyma attenuation was 37 HU in this case. An ROI area, as an example, is shown in the box. This patient died in hospital.

All included patients had both kidneys; they were evaluated by three radiologists (A.T. and S.T., with more than 10 years of experience in abdominal imaging and B.B., with more than 6 years of experience in abdominal imaging) blindly one from each other and with no access to patients’ clinical data. Three regions of interest (ROI) were positioned in every kidney, to include both the cortex and the medulla in the anatomical region as close as possible to the hilum [19,23]. In general, ROIs were placed slightly cranially to the hilum (Figure 1). ROIs areas were kept at a value between 0.5–0.7 cm^2^ to include cortex and medulla and excluding, as much as possible, hypodense caliceal and pelvic areas. The mean value of attenuation of the six ROIs (three for the right and three for the left) was recorded for subsequent analysis. The 128-slice-scanner was equipped with state-of-the-art automatic tube voltage selection (ATVS) algorithms for automatic selection of kVp and mA settings to reduce radiation dose. The algorithms automatically selected the kVp, and mA settings to give an adequate contrast-to-noise ratio (CNR) at the lowest volume CT dose index (CTDIvol). The 16-slice and 64-slice-scanners had tube voltage 120 kV, tube current 120 mA with mA modulation (64 × 0.625-mm collimation and 1.375 pitch). Axial images were reconstructed with at least 5.0-mm slice thickness and 5.0-mm interval. In addition, the presence or absence of perirenal fat stranding (PFS), defined as linear or curvilinear soft-tissue attenuations in the perirenal area (Figure 1), was recorded as a binary variable. All discrepancies among radiologists were resolved in consensus.

### 2.3. Statistical Analysis

We categorized the cohort into quartiles of renal parenchyma attenuation (RPA). Normally distributed variables are presented as mean ± SD and compared using an independent or paired *t*-test, as appropriate. Nonparametric continuous variables are presented as median with interquartile range (IQR). Comparisons between groups were made by analysis of variance. Comparisons of proportions were made using the χ^2^-test or Fisher’s exact test, as appropriate. Missing values were below 5%. To identify whether CT renal RPA and/or PFS were associated with the development of AKI, logistic regression analyses were performed. A multivariable model was used to adjust for other risk factors significantly associated with AKI, RPA and PFS.

Time to event analyses on mortality were performed using: (i) Kaplan–Meier method for survival curves estimation and log-rank test to compare them; (ii) univariate and multivariate Cox regression models. Hazard ratios (HR) along with their 95% confidence intervals (CI) were reported. Covariates included all available clinical variables with biological plausibility and showing a *p*-value at univariate analysis <0.10. The time variable was defined as the interval time between baseline date (the day of the first respiratory sample positive for SARS-CoV-2) and the date of endpoint occurrence or the last available follow-up.

## 3. Results

### 3.1. Clinical Characteristics on the Basis of Kidney Attenuation at Chest CT

During the study period, 854 patients received a diagnosis of COVID-19 either upon hospital admission or during hospitalization. 777 patients had available data about previous kidney function within the 180 days before COVID-19 diagnosis, necessary to define AKI. Of 777 patients, 134 underwent chest CT during hospitalization. The final study population consisted of 86 patients, because of the exclusion of patients with enhanced CT, urolithiasis, renal tumors, or respiratory artifacts altering kidney attenuation values (Figure S1). The main clinical characteristics of the study population as a whole and on the basis of RPA quartiles (Q1 vs. Q2–Q4) are shown in Table 1.

The mean age was 68 ± 13 years, 74.4% were males, with a proportion of CKD and diabetes of 20.9 and 12.8%, respectively. Patients showed an admission mean creatinine of 1.0 (4.0) mg/dl and eGFR of 70 ± 26 mL/min. 21% (n = 18) of patients had CKD and 38% (n = 33) developed AKI.

Despite a similar distribution of demographic and laboratory characteristics, and a similar prevalence of comorbidities, data reported in Table 1 show that patients with a CT RPA below 24 HU (first quartile, Q1) had less frequent pre-existing renal damage (4 vs. 27%, *p* = 0.022) and were more likely to develop AKI when compared with patients with higher RPA (χ^2^ = 2.77, *p* = 0.014). This independent relationship was confirmed with multivariate analysis (OR 3.88, 95% CI [1.17–12.92], *p* = 0.027; Table S1).

### 3.2. Clinical Characteristics on the Basis of Perirenal Fat Stranding at Chest CT

Patients with PFS (n = 59) were older and were more likely to be male, with a positive history of hypertension and clinical signs of renal damage (Table 2).

Nevertheless, with multivariate analysis the relationship between creatinine levels and PFS was lost (Table S2).

### 3.3. Determinants of AKI

The logistic regression analysis to assess the relationship between AKI and other variables, includes the parameters of critical illness score, estimating the severity of COVID-19 infection [24]. At multivariate logistic regression analysis, being in the first quartile of CT RPA was associated with a four times higher risk of AKI, (OR 4.56 CI [1.27–16.44], *p* = 0.020) after adjustment for age, gender, hypertension, kidney function at admission and other comorbidities, as recorded in Table 3.

### 3.4. Kidney Imaging According with Kidney Function as Predictor of Outcome

During a mean 22 ± 15 days of admission, 32 patients died (37.2%). The occurrence of death during the 9 months follow up was 41%.

**Figure 2:**
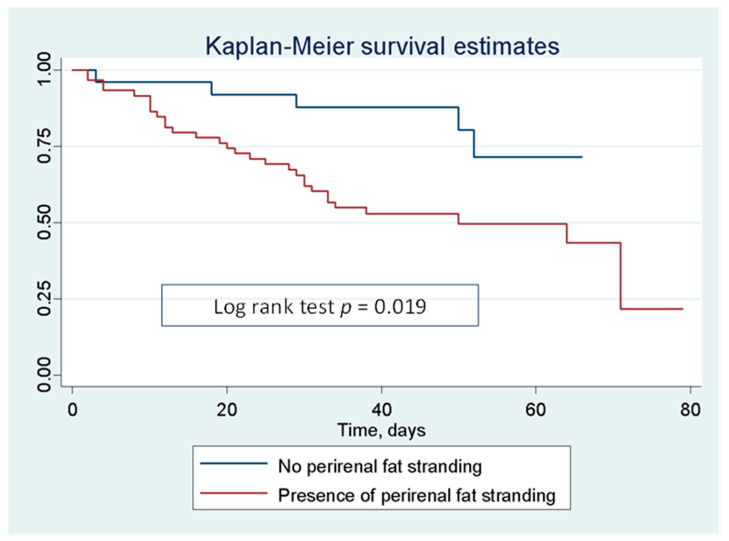
Kaplan–Maier curves of survival for intra-hospital death of hospitalized COVID-19 patients on the basis of the presence of CT perinephric fat stranding (PFS).

Patients with CT PFS had a higher risk of intra-hospital (Figure 2) and 9-month (Supplementary Figure S2) mortality compared with patients without this radiological sign (Log rank test *p* = 0.019, Figure 2).

Patients with lower values of RPA on the chest CT (first quartile, <24 HU) seemed to be at a higher risk of death compared to patients without it, but the result did not reach statistical significance (Supplementary Figure S3).

Table 4 shows that creatinine levels, the occurrence of AKI and the presence of PFS are significantly associated to intra-hospital mortality. In contrast, the presence of CKD and low RPA were not significantly associated with death in COVID-19. In the multivariate analysis the risk of mortality increased by almost three times for the occurrence of AKI, and by four times for the presence of PFS, even after including age, gender, kidney and heart function as covariates (HR 2.71 [95% CI 1.21–6.08, *p* = 0.015], and HR 4.14 [95% CI 1.16–14.79, *p* = 0.029], respectively).

## 4. Discussion

The main findings of this study were (1) that demonstration of low RPA on an unenhanced CT can detect COVID-19 patients who have a four times higher risk of developing AKI independently of baseline renal function and possible confounding factors and (2) that the presence of PFS can identify patients with higher mortality risk.

AKI is a common complication affecting 5–37% of hospitalized patients with COVID-19 [7,25,26] and is associated with high mortality [5,7]. Currently, the exact mechanisms of AKI in COVID-19 patients are unclear. Because of this, the latest research seeks to identify potential mechanisms of COVID-19–related AKI [26,27,28,29,30], which would allow for potential interventions to reduce this devastating complication.

Imaging is of utmost importance in detecting and monitoring lung impairment caused by SARS-CoV-2 infection. CTs are commonly acquired in patients admitted to hospital for COVID-19, either with or without contrast media, according to their clinical indications. Using dual-energy computed tomography, several patients with mild-to-moderate COVID-19 showed kidney perfusion defects possibly suggestive of small areas of cortical necrosis due to micro-thrombosis at cortical level [15]. We explored the clinical significance of a “smart” assessment of renal parenchyma and PFS on non-enhanced CT.

Patients with an RPA belonging to the first quartile (i.e., <24 HU) were four times more likely to have AKI when compared with other patients. We also found that this association was not related to other factors such as age, sex or gender, serum creatinine and other comorbidities. The mechanism explaining this relationship is not clear; we could assume that elevated intrarenal pressure due to microthrombosis and inflammation determine an increase of intrarenal fluid consistent with reduced RPA onCT. Moreover, we observed an increased risk of death in subjects in the lower quartile of RPA. This data, even if not reaching statistical significance, suggests a trend that could be confirmed in larger studies.

In our patients, we observed a direct relationship between creatinine levels at admission and the presence of PFS at univariate analysis (Table 3). PFS can be encountered in a wide spectrum of diseases and conditions including acute ureteral obstruction, pyelonephritis, acute pancreatitis and was found to be an independent prognostic factor of cancer-specific survival in ureteral urothelial carcinoma [31]. We expanded these data describing the increased risk of intra-hospital mortality in COVID-19 patients showing PFS on non-enhanced CT.

Altogether, our data suggest that in the context of greater global risk, as it is the case of COVID-19 hospitalization, kidney imaging can reach clinical significance. In particular, RPA could help identifying patients at risk for AKI and PFS might recognize those patients who are more prone to unfavorable outcomes (Figure 2 and Table 4). The development of PFS in COVID-19 patients is not clear; one possible explanation is that the perirenal space with the reticular bridging septa connecting the renal capsule to the Gerota’s fascia is affected by a COVID-19 related inflammatory response similarly to what happens in other inflammatory conditions affecting the kidney such as pyelonephritis and acute pancreatitis even in patients without obstruction. Another possible mechanism could be the presence of elevated intrarenal pressure, due to microthrombosis, draining backwards to the perinephric lymphatics running along the fibrous septa of the perirenal space. This hypothesis could explain why PFS on CT scans is associated with poor prognosis in COVID-19.

This study has several limitations. First, there was a relatively low number of cases due to the inclusion criteria (hospitalization, known renal function and unenhanced chest CT). Then, the retrospective nature of the data collection, which did not allow a perfect timing between CT and biochemical data, introduced potential biases. Moreover, while none of the CT scans showed signs diagnostic for pyelonephritis, we cannot establish the exact prevalence of urinary tract infection in our study patients. In this setting, the possibility to find some statistically significant data, especially regarding the association between RPA and mortality, was reduced, despite the strict inclusion criteria applied. Moreover, we are not aware if these results will be reproducible with other COVID-19 variants and in patients who received vaccination (any number, type and timing). Studies on vaccinated patients will clarify whether the low RPA and the presence of PFS can be considered predictive tools also in this context.

Among the strengths, this study has been conducted in a real-life scenario occurring in the first pandemic wave, and the diagnosis of AKI and the presence of pre-existing CKD are based on the mean of the values of creatinine available in the 180 days prior to admission for each of the patients recruited. While the sample size is relatively small in comparison to COVID-19 patients who often get CT studies, to our knowledge this is the largest cohort of patients in which kidney imaging was correlated with complete clinical parameters and follow-up.

## 5. Conclusions

In conclusion, our study provides for the first time, a framework for opportunistic evaluation of renal parenchyma in patients who undergo unenhanced CT on admission for COVID-19, showing that low RPA values (i.e., below 24 HU) are independent predictors of AKI development. This “smart” measure, could help physicians identifying patients with a higher risk of AKI without the need of historical data and independently by parameters of renal function frequently unavailable in an ICU setting. Accordingly, the detection of PFS, identifying more frail subjects, could help physicians in defining more appropriate diagnostic and therapeutic strategies. These findings call for more research studies on the link between CT kidney imaging and renal impairment in COVID-19 patients.

## Figures and Tables

**Table 1:** Baseline characteristics of the study cohort on the basis of Renal Parenchyma Attenuation (RPA).

	All	Q1 (<24)	Q2-Q3-Q4 (≥24)	*p* Value
Variables				
N	86	23	63	
RPA, HU	31.9 ± 23.1	16.4 ± 6.5	37.6 ± 24.3	<0.001
Demographic Characteristics				
Age, years	68 ± 13	65 ± 12	68 ± 13	0.295
Male gender, %	74.4	69.6	76.2	0.533
Hypertension, %	42.3	47.8	40.3	0.534
Diabetes, %	12.9	8.7	14.5	0.478
Chalson score	1.4 ± 2.1	1.3 ± 2.0	1.4 ± 2.1	0.871
Systolic blood pressure, mmHg	133 ± 23	131 ± 26	134 ± 22	0.673
Diastolic blood pressure, mmHg	74 ± 14	74 ± 15	75 ± 14	0.846
Heart rate, beats per min	88 ± 17	90 ± 19	87 ± 18	0.546
Respiratory rate, n/min	21 ± 7	21 ± 7	21 ± 7	0.772
Glasgow coma scale	14.1 ± 2.7	13.6 ± 3.7	14.3 ± 2.3	0.271
O_2_ saturation, %	91 ± 9	92 ± 8	90 ± 10	0.570
PaO_2_/FiO_2_ ratio	250 ± 94	222 ± 87	258 ± 98	0.277
Temperature, °C	37.6 ± 1.1	37.4 ± 0.8	37.6 ± 1.1	0.456
Laboratory Characteristics				
White blood cell count, x10^9^/L	8865 ± 6447	9153 ± 5700	8760 ± 6739	0.804
Hemoglobin, g/dL	13.1 ± 2.1	13.2 ±2.3	13.1 ± 2.1	0.889
Platelets count, ×10^9^/L	215 ± 101	224 ± 121	212 ± 93	0.606
Creatinine, mg/dL	1.0 (4.0)	0.9 (0.1)	1.0 (0.5)	0.111
eGFR, ml/min/1.73 m^2^	71.2 (37.7)	73.3 (31.8)	68.7 (38.3)	0.100
Urea, mg/dL	58 ± 42	47 ± 25	62 ± 46	0.156
Alanine aminotransferase, U/L	41 ± 30	43 ± 31	40 ± 29	0.733
D-dimer, mg/L	1067 (959)	1312 (574)	1939 (448)	0.159
Creatine kinase, U/L	98 (119)	130 (118)	89 (84)	0.723
Lactate dehydrogenase, U/L	285 (201)	317 (261)	280 (207)	0.545
C-reactive protein, mg/L	89 (112)	101 (179)	83 (109)	0.149
Interleukin-6, pg/mL	54 (90)	57 (89)	49 (76)	0.084
Procalcitonin, ng/mL	1.08 ± 5.39	0.66 ± 1.02	1.25 ± 6.39	0.665
Potassium, mEq/L	3.8 ± 0.6	3.6 ± 0.5	3.9 ± 0.6	0.080
Kidney Status				
Chronic Kidney Disease, %	20.9	4.3	27.0	0.022
Acute Kidney Injury, %	38.4	52.2	33.3	0.014
Acute Kidney Injury stages 1-2-3, %	13-9-16	9-9-35	14-9-9	0.046
Teraphy				
Hydroxychloroquine, %	94.4	95.2	94.1	0.850
Tocilizumab, %	48.3	67.7	41.9	0.111
Corticosteroids, %	98.0	100.0	97.5	0.596
Radiologic Findings in Chest CT				
Perirenal stranding, %	69.4	54.5	74.6	0.079
Bilateral pulmonary infiltration, %	43.0	47.8	41.3	0.587
Ground glass opacities, %	76.4	91.3	71.4	0.053
Honeycombing opacities, %	9.4	9.1	9.5	0.952
Outcomes				
Hospitalization, days	22 ±15	26 ±12	21 ±16	0.219
Intra-hospital mortality	37.2	43.5	34.9	0.467
Follow up, days	242 ±174	213 ±179	255 ±172	0.350
9 months-mortality, %	41.9	52.2	38.1	0.241

Data presented as mean ± standard deviation, or median (IQR) or percentage. Abbreviations: IQR, Interquartile Range; PaO_2_, arterial oxygen partial pressure; FiO_2_, fractional inspired oxygen; eGFR, estimated glomerular filtration rate, RPA, renal parenchyma attenuation; HU, Hounsfield units. RPA quartiles: Q1 (RPA −3, 23), Q2 (RPA 24, 28), Q3 (RPA 29, 34), Q4 (RPA 35, 173).

**Table 2:** Baseline characteristics of the study cohort on the basis of the presence of perirenal fat stranding (PFS).

	All	NO PFS	PFS	*p* Value
Variables				
N	86	27	59	
Demographic Characteristic				
Age, years	68 ± 13	61 ± 15	70 ± 11	0.003
Male gender, %	74.4	57.7	83.0	0.012
Hypertension, %	42.9	23.1	51.7	0.014
Diabetes, %	12.8	3.8	17.2	0.092
Charlson score	1.4 ± 2.1	1.1 ± 2.2	1.5 ± 2.1	0.469
Systolic blood pressure, mmHg	133 ± 23	129 ± 19	134 ±24	0.426
O_2_ saturation, %	91 ± 9	90 ± 8	91 ± 10	0.698
PaO_2_/FiO_2_ ratio	250 ± 94	249 ± 108	251 ± 96	0.948
Temperature, °C	37.6 ± 1.1	37.6 ± 1.1	37.5 ± 1.0	0.636
Laboratory Characteristics				
Creatinine, mg/dL	1.0 (4.0)	0.9 (0.4)	1.1 (0.5)	0.027
eGFR, ml/min/1.73 m^2^	71 (38)	82 (30)	65 (38)	<0.001
Urea, mg/dL	58 ± 42	43.2 ± 23.2	64.2 ± 45.6	0.039
C-reactive protein, mg/L	89 (112)	120 (181)	83 (101)	0.352
Interleukin-6, pg/mL	54 (90)	56 (84)	52 (83)	0.167
Procalcitonin, ng/mL	1.08 ± 5.39	0.51 ± 0.74	1.35 ± 6.52	0.192
Kidney Status				
Chronic Kidney Disease, %	20.9	11.5	25.4	0.149
Acute Kidney Injury, %	38.4	34.6	40.7	0.597
Acute Kidney Injury stages 1-2-3, %	13-9-16	8-11-15	15-8-17	0.774
Radiologic Findings in Chest CT				
Renal parenchymal attenuation, HU	31.9 ± 23.1	29.3 ± 17.6	33.3 ± 25.3	0.459
Bilateral pulmonary infiltration, %	43.0	34.6	47.5	0.271
Ground glass opacities, %	76.4	73.1	78.0	0.624
Honeycombing opacities, %	9.4	8.0	10.1	0.757
Outcomes				
Hospitalization, days	22 ± 15	22 ± 17	22 ± 15	0.986
Intra-hospital mortality	37.2	15.4	45.8	0.007
Follow up, days	242 ± 174	325 ± 131	209 ± 179	0.004
9 months-mortality, %	41.9	19.2	50.8	0.006

Data presented as mean ± standard deviation, or median (IQR) or percentage. Abbreviations: IQR, Interquartile Range; CKD, chronic kidney disease; PaO_2_, arterial oxygen partial pressure; FiO_2_, fractional inspired oxygen; eGFR, estimated glomerular filtration rate, RPA, renal parenchyma attenuation; HU, Hounsfield units.

**Table 3:** Univariate and multivariate logistic regression analyses for AKI in hospitalized patients with COVID-19.

	Univariate Model			Multivariate Model		
	Odds Ratio	95% CI	*p*	Odds Ratio	95% CI	*p*
Age, years *	1.00	0.97–1.03	0.960	0.96	0.91–1.01	0.146
Male gender *	0.87	0.32–2.33	0.777	0.26	0.05–1.23	0.090
Charlson index *	1.11	0.90–1.38	0.325	1.16	0.89–1.53	0.266
Hypertension	2.79	1.13–6.88	0.025	1.25	0.30–5.30	0.759
Diabetes	0.56	0.14–2.29	0.422			
Heart failure	1.19	0.25–5.70	0.828			
Creatinine, mg/dl	4.84	1.23–29.13	0.026	50.7	4.62–556.01	0.001
RPA <24 HU	2.22	0.86–5.74	0.100	4.56	1.27–16.44	0.020
Perirenal stranding	1.29	0.50–3.38	0.593			
O_2_ Saturation, %	0.98	0.93–1.03	0.479			
PaO_2_/FiO_2_ ratio	0.99	0.99–1.00	0.071			
Chest radiographic abnormality *	0.66	0.21–2.03	0.469			
Cancer history *	0.81	0.40–1.66	0.565			
Lactate dehydrogenase, U/L *	1.00	0.99–1.00	0.114			
Direct bilirubin, *	2.40	0.61–9.40	0.207			
L/N ratio *	0.69	0.21–2.26	0.545			
Dyspnea *	1.02	0.42–2.48	0.966			

Abbreviations: CI, confidence intervals; RPA, renal parenchyma attenuation; HU, Hounsfield units; L, lymphocytes; N, neutrophils. * variables included in clinical risk score to predict the occurrence of critical illness in hospitalized patients with COVID-19 [23].

**Table 4:** Univariate and multivariate Cox regression analyses for mortality in hospitalized patients with COVID-19.

Risk Factors	Univariate Model			Multivariate Model		
	HR	95% CI	*p*	HR	95% CI	*p*
Age, years	1.04	1.01–1.06	0.003	1.00	0.96–1.04	0.944
Male gender	2.74	1.16–6.46	0.021	1.78	0.68–4.59	0.266
Charlson index	1.11	0.95–1.30	0.168			
Hypertension	1.71	0.85–3.42	0.130			
Diabetes	1.52	0.58–3.95	0.390			
Chronic kidney disease	1.19	0.52–2.77	0.677	1.28	0.42–3.94	0.661
Heart failure	2.88	1.10–7.57	0.031	2.80	0.88–8.88	0.080
Creatinine, mg/dl	1.46	1.18–1.80	<0.0001	1.10	0.73–1.66	0.647
Acute kidney injury	2.00	1.03–3.89	0.042	2.71	1.21–6.08	**0.015**
RPA ≤24 (Q1)	1.52	0.74–3.12	0.249	1.44	0.63–3.29	0.381
Perirenal fat stranding	2.95	1.14–7.63	0.026	4.14	1.16–14.79	**0.029**
O_2_ Saturation, %	0.97	0.94–1.01	0.210			
PaO_2_/FiO_2_ ratio	1.00	0.99–1.01	0.178			
Chest radiographic abnormality *	1.23	0.48–3.18	0.668			
Cancer history *	1.53	1.01–2.33	0.043	1.40	0.86–2.27	0.176
Lactate dehydrogenase, U/L *	1.00	1.00–1.00	0.094			
Direct bilirubin, *	1.79	0.70–4.58	0.225			
L/N ratio *	0.84	0.44–1.62	0.611			
Dyspnea *	0.94	0.48–1.85	0.867			

Abbreviations: CI, confidence intervals; RPA, renal parenchyma attenuation; HU, Hounsfield units, Q1, first quartile of RPA; L, lymphocytes; N, neutrophil. * variables included in clinical risk score to predict the occurrence of critical illness in hospitalized patients with COVID-19 [23].

## Data Availability

The data presented in this study are available on request from the corresponding author. The data are not publicly available due to privacy.

## Supplementary Materials

The following supporting information can be downloaded at: https://www.mdpi.com/article/10.3390/jcm12030929/s1, Figure S1: Flow diagram for selection of study patients.; Figure S2: Kaplan–Maier curves of survival for 9 months of hospitalized COVID-19 patients on the basis of the presence of CT perinephric fat stranding (PFS); Figure S3: Kaplan–Maier curves of survival for hospitalized COVID-19 patients on the basis of AKI-specific cut off of Renal Parenchyma Attenuation (RPA); Table S1: Univariate and multivariate logistic regression analyses for the presence of Renal Parenchyma Attenuation (RPA) below 24 HU in CT of hospitalized patients with COVID-19; Table S2: Univariate and multivariate logistic regression analyses for the presence of perirenal fat stranding (PFS) in CT of hospitalized patients with COVID-19.
