# Supplementary material for: Role of Renal Parenchyma Attenuation and Perirenal Fat Stranding in Chest CT of Hospitalized Patients with COVID-19
Source: J Clin Med. 2023 Jan 25;12(3):929. doi: 10.3390/jcm12030929 (PMC9918001; doi:10.3390/jcm12030929)
Supplement: Supplementary file 1 [file jcm-12-00929-s001.zip › jcm-2147725-supplementary.pdf]

Supplementary Materials

Supplementary Table S1. Univariate and multivariate logistic regression analyses for the presence of Renal Parenchyma Attenuation (RPA) below 24 HU in CT of hospitalized patients with COVID-19.

	UNIVARIATE MODEL			MULTIVARIATE MODEL		
	**Odds Ratio**	**95% CI**	**P**	**Odds Ratio**	**95% CI**	**p**
**Age, years**	0.98	0.94–1.02	0.293			
**Male gender**	1.40	0.48–4.04	0.534			
**Charlson index**	0.98	0.76–1.26	0.869			
**Hypertension**	1.36	0.52–3.55	0.535			
**Diabetes**	0.56	0.11–2.81	0.482			
**Chronic kidney disease**	1.22	0.15–0.98	0.048	0.24	0.02–2.32	0.218
**Creatinine, mg/dl**	0.31	0.08–1.24	0.099	0.38	0.06–2.38	0.300
**Acute kidney injury**	1.08	0.82–5.77	0.105	3.88	1.17–12.92	**0.027**
**Ground glass opaticities**	4.20	0.89–19.8	0.070	3.78	0.73–19.6	0.113
**Perirenal fat stranding**	0.41	0.15–1.12	0.083	0.49	0.15–1.60	0.241

Abbreviations: CI, confidence intervals; HU, Hounsfield units.

Supplementary Table S2. Univariate and multivariate logistic regression analyses for the presence of perirenal fat stranding (PFS) in CT of hospitalized patients with COVID-19.

	UNIVARIATE MODEL			MULTIVARIATE MODEL		
	**Odds Ratio**	**95% CI**	**P**	**Odds Ratio**	**95% CI**	**p**
**Age, years**	1.06	1.01–1.10	**0.006**	1.04	0.99–1.09	0.073
**Male gender**	3.59	1.28–10.10	**0.015**	2.14	0.63–7.28	0.220
**Charlson index**	1.10	0.85–1.41	0.466			
**Hypertension**	3.57	1.25–10.18	**0.017**	1.49	0.43–5.14	0.531
**Diabetes**	5.10	0.61–42.14	0.130			
**Heart failure**	2.54	0.29–22.34	0.401			
**Creatinine, mg/dl**	12.48	1.95–79.65	**0.008**	4.86	0.74–31.63	0.098
**Acute kidney injury**	1.29	0.50–3.38	0.598			
**RPA <24 HU**	1.01	0.98–1.03	0.466			
**Chronic kidney disease**	2.61	0.68–9.96	0.159			

Abbreviations: CI, confidence intervals; RPA, renal parenchyma attenuation; HU, Hounsfield units.

Supplementary Figure S1. Flow diagram for selection of study patients.

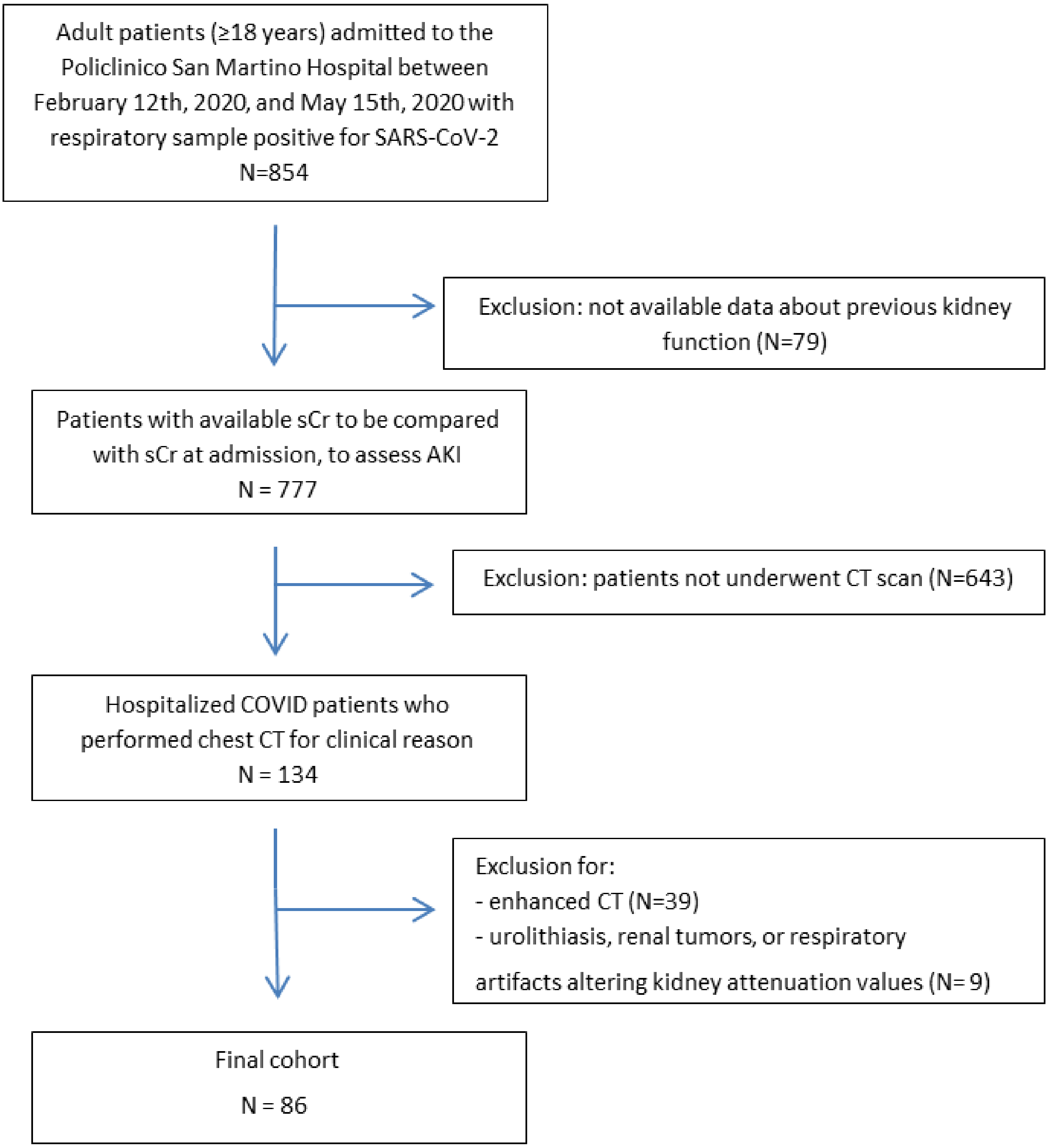

Abbreviations: sCr, serum creatinine; AKI, acute kidney injury; CT, computed tomography.

Supplementary Figure S2. Kaplan Maier curves of survival for 9 months death of hospitalized COVID-19 patients on the basis of the presence of CT perinephric fat stranding (PFS).

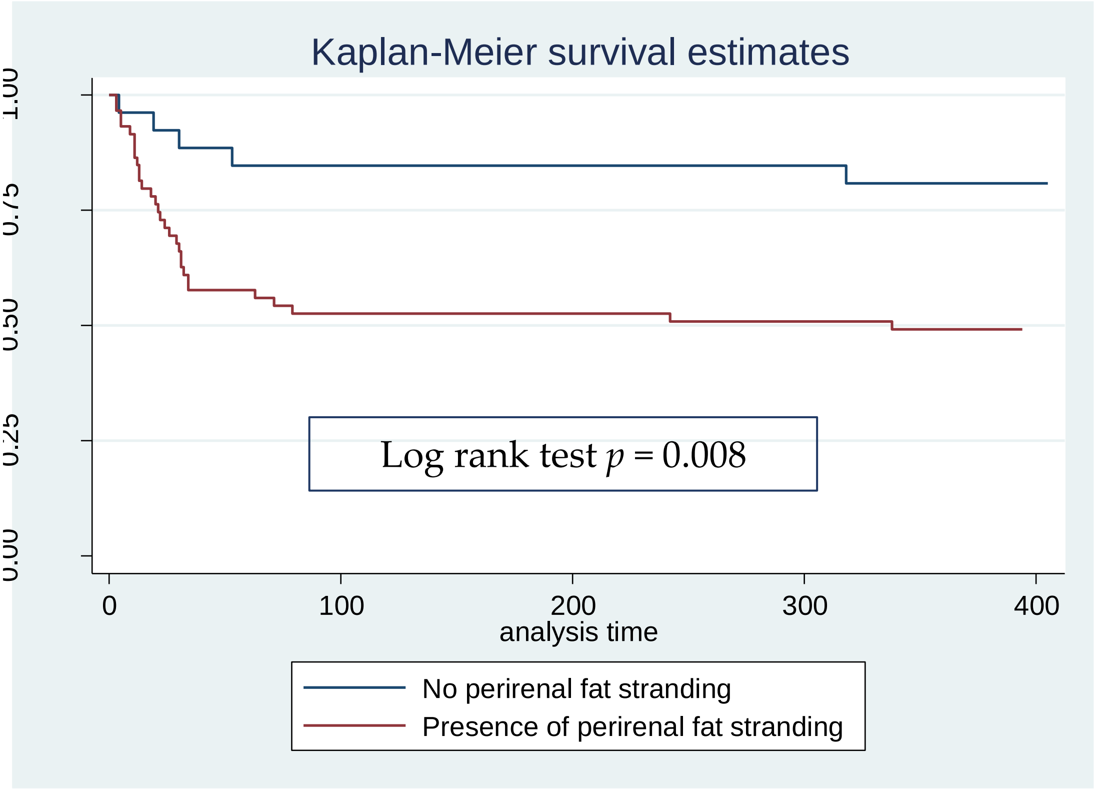

Supplementary Figure S3. Kaplan Maier curves of survival for hospitalized COVID-19 patients on the basis of AKI-specific cut off of Renal Parenchyma Attenuation (RPA).

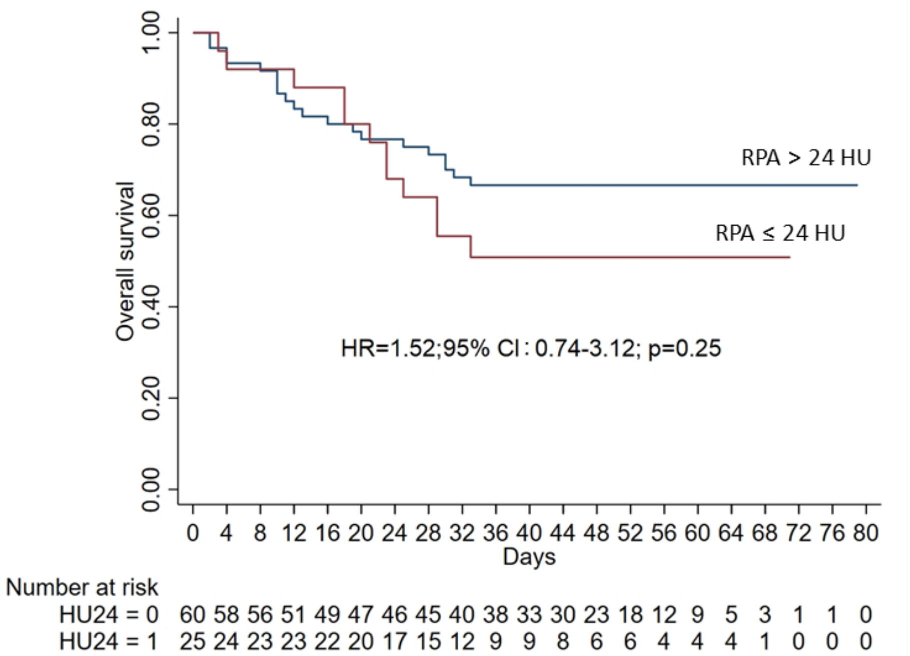

Abbreviations: HR, hazard ratio; CI, confidence intervals; RPA, renal parenchyma attenuation; HU, Hounsfield units.
